# Provider Driven Follow-Up in a Chest Pain Accelerated Diagnostic Protocol: Round Two of the PDSA Cycle in a Multidisciplinary Quality Improvement Patient Safety Project

**DOI:** 10.51894/001c.11727

**Published:** 2020-01-30

**Authors:** Isaac Troiano, Mary Mitchell, Mark Schury, Nikolai Butki

**Affiliations:** 1 Emergency Medicine McLaren Oakland; 2 Family Medicine McLaren Oakland

**Keywords:** chest pain, multidisciplinary, adp, qips

## Abstract

**CONTEXT:**

In 2016, the McLaren Oakland Department of Emergency Medicine developed and implemented a Chest Pain Accelerated Diagnostic Protocol (CP-ADP) to identify patients presenting to the emergency department (ED) with chest pain who were at low risk for acute coronary syndrome (ACS) and appropriate for outpatient follow-up. The evaluation of the QI/PS project demonstrated that only 47% of the patients discharged from the ED under the CP-ADP received outpatient follow-up. In response, a second round of the PDSA cycle modified the CP-ADP to add a multidisciplinary provider driven follow-up.

**METHODS:**

After ED discharge, patients in the CP-ADP with provider driven follow-up were contacted by a primary care physician to schedule a follow-up appointment. The premise was that this provider driven follow-up would alleviate navigation of the health care system as a barrier to follow-up.

**RESULTS:**

The evaluation of the modified CP-ADP with provider driven follow-up demonstrated that 9 of the 30 patients discharged from the ED were able to be contacted. 21 of the patients were unable to be reached by the phone number they provided. Only 3 patients discharged with provider driven follow-up showed up to follow up appointments.

**CONCLUSIONS:**

There were some internal process failures identified that contributed to the low numbers of patients that were successfully contacted. External factors such as patient access to phones and means of communication were also discussed as factors that were originally not considered.

## INTRODUCTION

In 2016, the McLaren Oakland Department of Emergency Medicine in Pontiac, MI developed and implemented a Chest Pain Accelerated Diagnostic Protocol (CP-ADP) using the HEART score as a decision-making tool. The protocol uses history, EKG interpretation, age, risk factors and troponin to calculate a HEART Score. Based on evidence from other published protocols, it was determined that a HEART score of 3 or less stratified a patient as low risk for acute coronary syndrome (ACS) and appropriate for outpatient follow-up.^1–3^ Of the 30 patients discharged under the CP-ADP, 14 (47%) obtained outpatient follow-up within seven days.^4^

To identify possible reasons, the authors searched the literature which uncovered numerous studies addressing the discontinuity of care between ED and primary care physicians (PCPs). One study, for example, identified differing expectations regarding follow-up timeframe between emergency physicians (EPs) and PCPs.^5^ Another study identified limited impact of discharge instruction on patients’ compliance with follow-up with PCPs or decrease in ED utilization.^6^ In addition, lack of insurance and PCP appointment availability have also been identified as causes of noncompliance with outpatient follow-up following emergency department treatment.^7–12^

**Figure 1:**
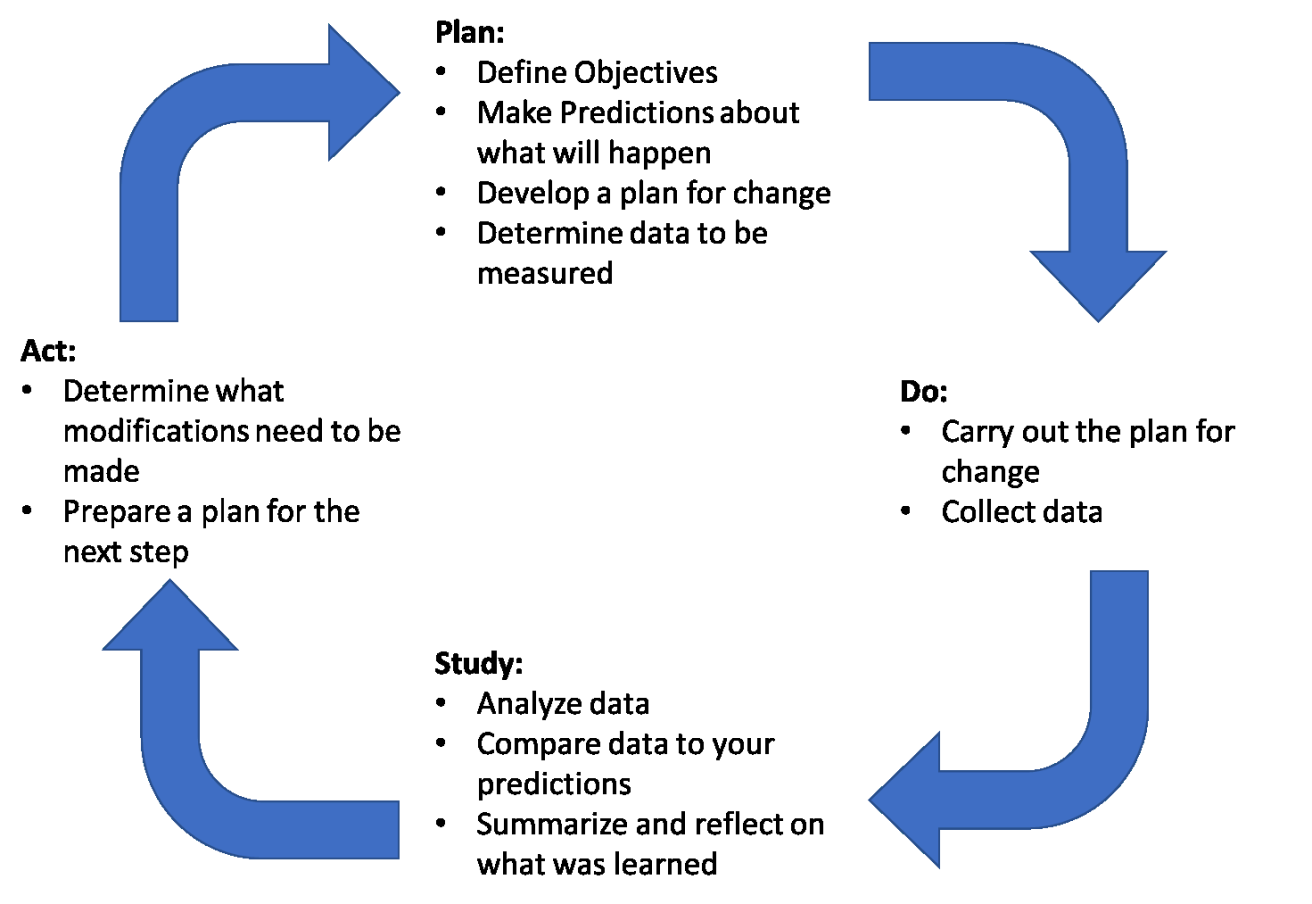
The Plan-Do-Study-Act (PDSA) Cycle http://www.ihi.org/resources/Pages/HowtoImprove/ScienceofImprovementTestingChanges.aspx

The overlying theme in these studies is that the current health care system is difficult for patients to navigate. To alleviate these barriers, the authors initiated a second round of the Plan-Do-Study-Act (PDSA) cycle.^13^ (Figure 1) in 2018 and modified the CP-ADP to include a multidisciplinary provider driven follow-up. Under the new CP-ADP with provider driven follow-up, patients discharged under the CP-ADP were given the option to have their contact information faxed to a PCP office who would contact the patient directly to schedule a follow-up appointment.

There were two primary outcomes for the CP-ADP with provider driven follow-up. The first was to increase the total number of patients successfully contacted after ED discharge. The second was to increase the total number of patients who had follow-up appointments within seven working days of discharge. Otherwise, the CP-ADP with provider driven follow-up used the same decision-making tool (HEART Score), the same determination of low risk (HEART Score less than or equal to 3) and the same patient discharge instructions as the original study.

## METHODS

**Figure 2:**
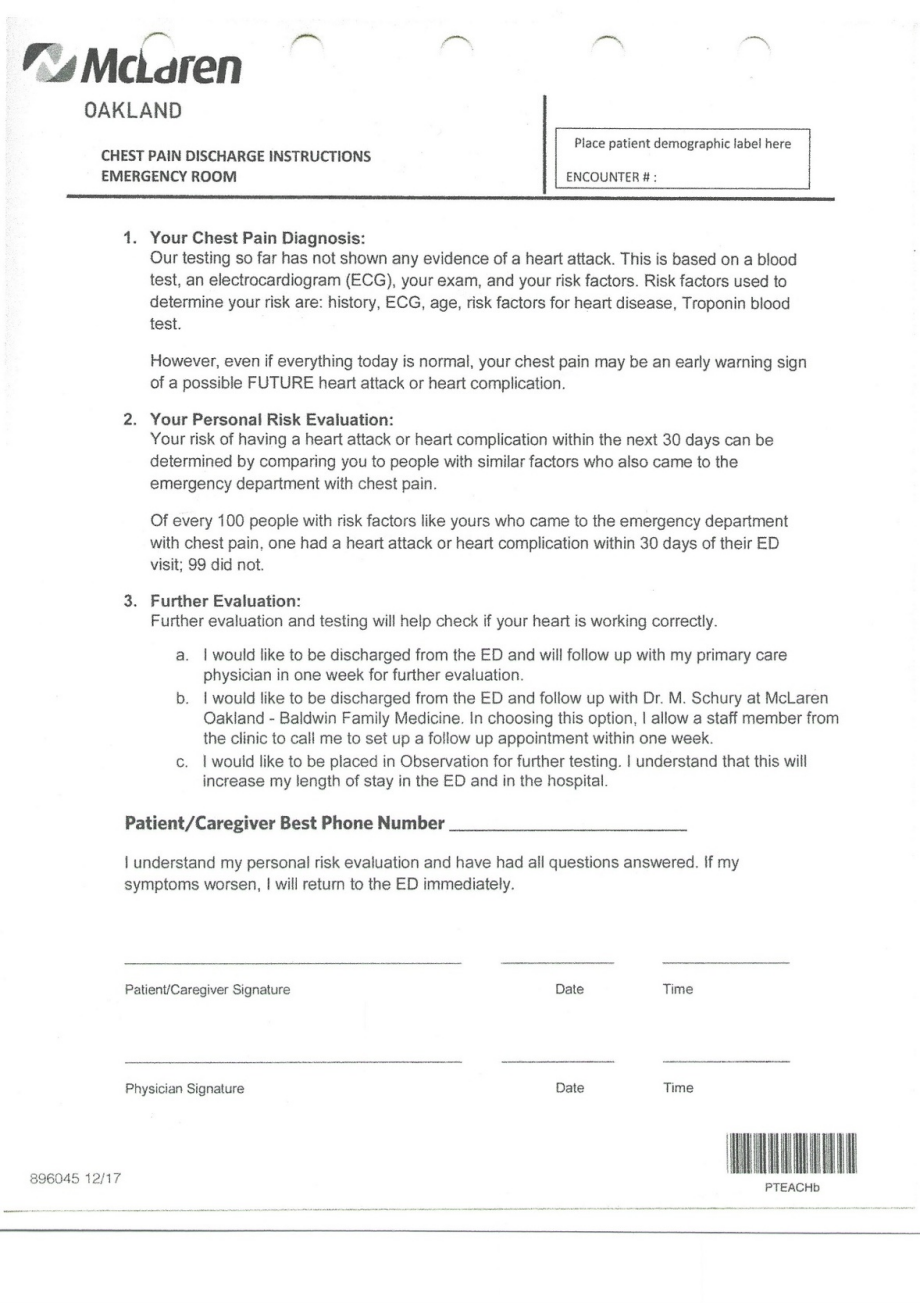
Chest Pain Accelerated Diagnostic Protocol (CP-ADP)

This study was determined to not qualify as human subject research and was not subject to IRB oversight by the McLaren Corporate IRB prior to any data collection. The McLaren Oakland Family Medicine Resident Clinic (Baldwin Clinic) served as the PCP office for the CP-ADP with provider-driven follow-up. The CP-ADP discharge document was unchanged from the prior CP-ADP^4,14^ except for the addition of a field where the patient consents to be contacted via provider-driven follow-up if they choose that discharge option. Also, a space was added on the discharge document for the patient to write their preferred contact phone number. (Figure 2)

The EM residents at McLaren Oakland serve as the primary source of communication with the patients and families during the ED visits and in the shared decision-making process when using the CP-ADP. After discussion of the HEART Score and CP-ADP, if the patient elected to be discharged from the emergency department with provider driven follow-up, the emergency medicine residents were charged with completing the CP-ADP discharge document with the patient and handing the CP-ADP discharge document to the ward clerk (also referred to as ‘unit coordinator’ or ‘HUC’). Three 15-minute education sessions were held for three consecutive months to train the emergency medicine residents on how to properly use the provider driven follow-up. Three separate sessions have been used at our institution to maximize the number ED residents trained in a new process.^15^ There was no change was made in the nursing discharge process. Two education sessions were provided to ward clerks regarding the fax process and the appropriate paperwork to include in the fax.

**Figure 3:**
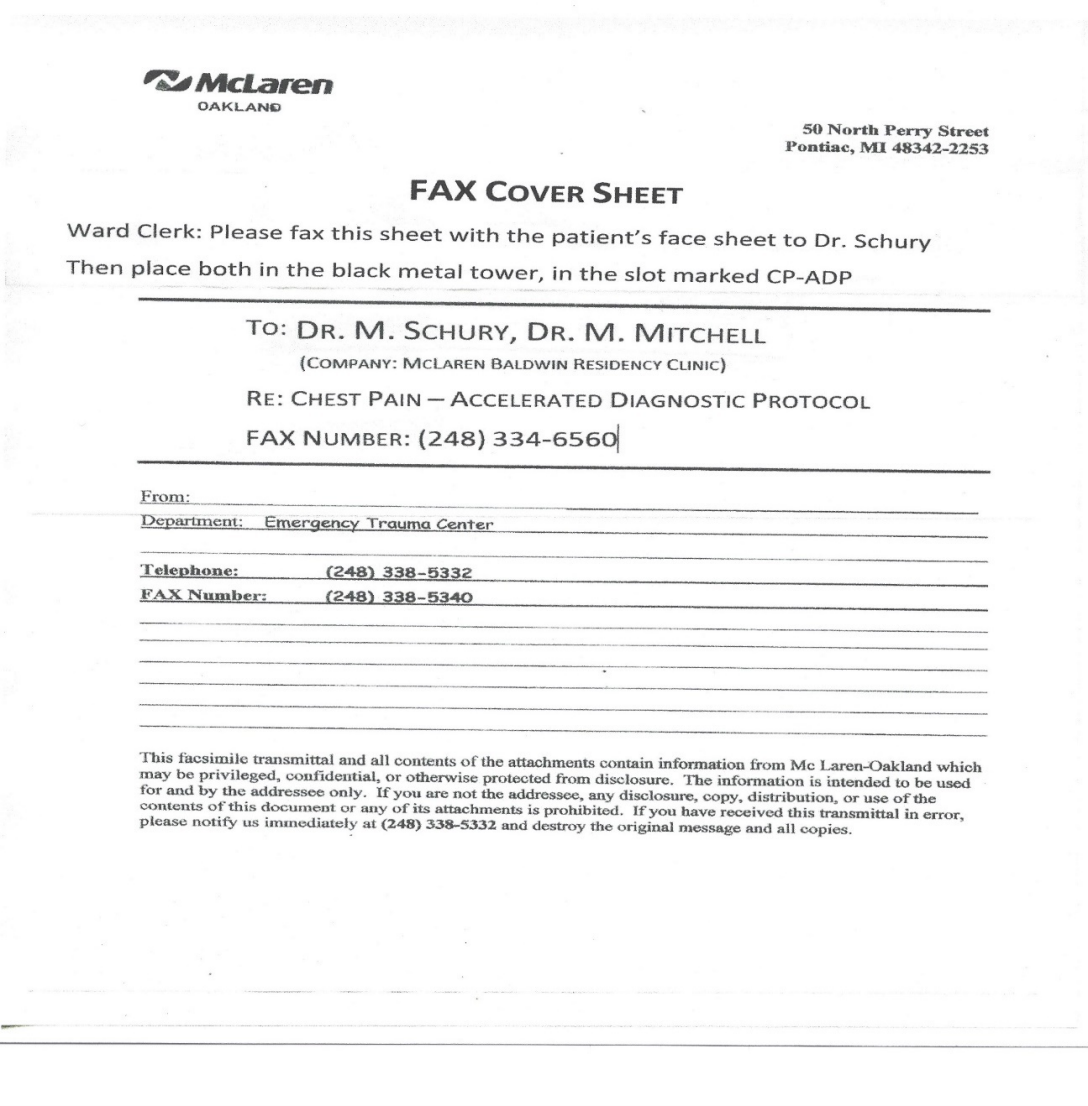
Chest Pain Accelerated Diagnostic Protocol (CP-ADP) Discharge Document

The ward clerks were charged with faxing the CP-ADP discharge document, a standardized fax face-sheet (Figure 3) and the patient information face sheet to the Baldwin Clinic after ED discharge.

Upon receipt of the fax, the Baldwin Clinic staff were instructed to place the paperwork in a specific file for provider-driven follow-up. Within 24 hours of receipt, the patients were contacted by the office manager who volunteered to be the contact person at the Baldwin Clinic. If a patient was an established patient at the Baldwin Clinic an appointment was scheduled for 3-5 days after their ED visit. If the patient was established with another PCP, the patient was encouraged to contact their doctor’s office to schedule a prompt appointment, if he/she hadn’t done so already. If a patient was not established with a PCP, the patient was provided information about the Baldwin Clinic and, with the patient’s permission, an appointment was scheduled at the Baldwin Clinic 3-5 days after their ED visit.

## RESULTS

During the evaluation period between October 2018 and March 2019, 50 patients discharged from the McLaren Oakland ED utilizing the CP-ADP requested provider driven follow-up. 30 (60%) patients’ discharge documentation was received at the Baldwin Clinic.

Of the 30 patients, Baldwin Clinic records confirmed that 7 (23%) patients were already established patients at the Baldwin Clinic. 23 (76%) were not established patients at the Baldwin Clinic.

Contact via telephone was attempted for all 30 patients. If there was a working phone number, three contact attempts were made with messages left. Attempts to contact were discontinued if there was a non-working phone number.  Nine (30%) of the 30 patients were contacted successfully and scheduled follow-up appointments. 21 (80%) patients were unable to be contacted using the phone number provided.

Of the nine patients successfully contacted, five patients were already established Baldwin Clinic patients. Four patients had established relationships with another PCP. These four patients verbally agreed to make an appointment to follow up with their PCP and were no longer followed.

Of the five established Baldwin Clinic patients 3 (10%) showed for their appointment. (Table 1)

**Table 1:** Summary of Discharge and Follow-Up Patient Subsets

	**N = 50**
**Discharge documentation received at Baldwin Clinic**	30 (60%)
*Established patients*	7 (23%)
*Not established patients*	23 (76%)
**Of those with documentation received at Baldwin Clinic**	**N = 30**
*Successful follow-up scheduled*	9 (30%)
established patients	5 (16.7%)
showed up to appointment	3 (10%)
relationship with another PCP	4 (13.3%)
*Unable to be contacted*	21 (80%)

## DISCUSSION

The goal of CP-ADP initiative was to increase the number of patients who followed up after ED discharge. The first CP-ADP put the responsibility to seek appropriate follow-up upon the patient which yielded disappointing results. Because many patients were not able to be contacted during the evaluation period, the authors were unable to determine reasons as to why patients were not following up. Therefore, the authors performed a literature search to elicit possible barriers. A common theme was that the medical system is difficult to navigate, even for the medically literate.^7–12^ This second round of the PDSA cycle for the CP-ADP aimed to remedy the lack of follow-up from the first study by eliminating placing the onus for follow-up on the patient.

During the study period, a significant portion of patients were lost due to internal system failures. Twenty (40%) patients requesting provider driven follow up did not have their paperwork appropriately delivered to the ward clerk or the ward clerk did not appropriately fax the required paperwork to the Baldwin Clinic. This was the second time the ED providers participated in a similar project and despite serial education sessions for all parties involved, the loss of patients due to system processes alone identified a vulnerability in the process. Even more recurrent multidisciplinary training in the form of short ‘booster’ training sessions may be needed to alleviate process breakdowns, as has been the need with prior QI/PS projects at our institution.^15^

There was also significant failure in contacting patients by telephone. Many patients served by the McLaren Oakland emergency department have inconsistent or transient housing. So, when designing the provider-driven follow-up, attempting to contact patients by mail was felt to be a futile option. Likewise, many patients do not have access to internet communication. So email contact was not deemed to be a reliable communication platform. Cell phone use anecdotally seems to be ubiquitous, even in the low-income populations such as those served by our emergency department.^16^ However, 21 of the 30 patients were not able to be contacted by telephone. The study did not measure how many phone numbers were inactive vs how many did not answer or respond to voicemail. While efforts were made to eliminate nonworking phone numbers by having the patient themselves provide a contact phone number on the discharge document, possibly a much larger issue was brought to light regarding patient resources. Consistent cell phone access is assumed but can be cost-prohibitive for many patients. Our ED serves a low-income, underserved population. It is possible that believing that all patients have access to communication resources is an unrealistic assumption.

## Conclusions/Limitations

Many patients presenting to the ED with chest pain can be safely discharged from the ED with outpatient follow-up. However, our institution has struggled developing a program that maximizes participation in a chest pain ADP and ensures appropriate outpatient follow-up. While unable to find studies examining comparable populations in a similar environment, the experience of the author as an EM clinician suggests that reasons for patients declining participation in the CP-ADP could include under-appreciating the importance for follow-up, under-appreciating the limitations of the ED workup to exclude coronary disease as a cause for their chest pain and lack of access to resources such as phones and internet to help navigate the health care systems. Future studies could aim to have one short question asking those declining to participate to specify why they decline the opportunity.

The results from this study could help future studies address the complex phenomenon of following up with patients who are likely to end up back in the ED if their cardiac needs are not optimally addressed in office-based settings. Future CP-ADP PDSA cycles should focus on identifying and overcoming deficiencies in communication resources that many of our patients have. Consideration for survey-based studies regarding communication resources and interest to personal health might help direct how best to cater to our patients’ needs and concerns.

### Conflict of Interest

The authors declare no conflict of interest.
